# N-terminal pro-B-type natriuretic peptide in amniotic fluid of fetuses with known or suspected cardiac load

**DOI:** 10.1371/journal.pone.0177253

**Published:** 2017-05-18

**Authors:** Christina Leufgen, Ulrich Gembruch, Birgit Stoffel-Wagner, Rolf Fimmers, Waltraut M. Merz

**Affiliations:** 1 Department of Obstetrics and Prenatal Medicine, University Bonn Medical School, Bonn, Germany; 2 Institute for Clinical Chemistry and Pharmacology, University Bonn Medical School, Bonn, Germany; 3 Institute for Medical Biometry, Informatics and Epidemiology, University Bonn Medical School, Bonn, Germany; Merck & Co., UNITED STATES

## Abstract

**Background:**

Myocardial dysfunction occurs in a variety of fetal disorders. Findings from adult cardiology, where n-terminal pro-B-type natriuretic peptide (nt-proBNP) is an established biomarker of left ventricular dysfunction have been extended to fetal life. Since fetal blood sampling is technically challenging we investigated amniotic fluid nt-proBNP for its suitability to diagnose fetal myocardial dysfunction.

**Methods:**

Ultrasound, Doppler examination and echocardiography was applied to classify cases and controls. Amniotic fluid nt-proBNP to amniotic fluid total protein ratio was calculated and compared to the gestational age-dependent reference intervals. In a subset of cases, fetal and maternal plasma nt-proBNP levels were determined.

**Results:**

Specimen from 391 fetuses could be analyzed (171 cases, 220 controls). There was a high correlation between amniotic fluid and fetal blood nt-proBNP levels (r = 0.441 for cases; r = 0.515 for controls), whereas no correlation could be detected between maternal and fetal (blood and amniotic fluid) nt-proBNP concentrations. Specificity and positive likelihood ratio of amniotic fluid nt-proBNP to amniotic fluid total protein ratio were high (0.97 and 4.3, respectively).

**Conclusion:**

Amniotic fluid nt-proBNP measurement allows diagnostic confirmation of fetal myocardial dysfunction. It may serve as a useful adjunct in addition and correlation to existing tests of myocardial function, particularly in the context of invasive fetal therapy, where access to the amniotic cavity is part of the procedure.

## Introduction

Myocardial dysfunction accompanies a variety of fetal conditions. These include not only structural cardiac defects, primary myocardial diseases and cardiomyopathies. Diseases which imply an increased cardiac output or altered intrathoracic volume relations (anemia, arterio-venous malformations, sacrococcygeal teratoma, congenital pulmonary airway malformation), chronic twin-transfusion syndrome (TTS) in monochorionic pregnancies, and fetal growth restriction (FGR) have also been shown to impact on cardiac function [1–8]. Minimally invasive surgical techniques have been developed for intrauterine therapy of some of the aforementioned conditions. Serial assessment and timely delivery remain the standard of care for the others [9–12]. Methods available for the assessment of fetal myocardial function include echocardiography, Doppler sonography of the fetal vasculature, myocardial tissue Doppler, and speckle tracking. The influence of cardiac loading conditions and the inter- and intraobserver variability limit the application of these image-based methods [13, 14].

In adult and pediatric patients with heart failure, the cardiac peptide hormone B-type natriuretic peptide (BNP) or its inactive cleavage product, n-terminal pro-B-type natriuretic peptide (nt-proBNP) is an established biomarker for the diagnosis and management of myocardial dysfunction as it allows a comprehensive assessment of the myocardial structure, function and loading. Nt-proBNP- guided therapy has been incorporated into clinical practice [15–20]. Cardiac myocytes secrete BNP in response to stretch; together with A-type natriuretic factor (ANF) BNP regulates pre- and afterload by increasing natriuresis and antagonizing the renin-angiotensin-aldosteron system. Additionally, BNP plays a central role in cardiac remodeling; it has been shown that knock-out mice develop myocardial fibrosis [21]. Recently, nt-proBNP has been analyzed in fetal blood samples, where its role as biomarker in fetal medicine could be established [22–29].

Fetal blood sampling is a technically challenging procedure. In comparison, amniocentesis has a higher success and a lower complication rate. Additionally, access to the amniotic cavity is gained for invasive intrauterine therapies. Difficulties in quantitative amniotic fluid (AF) analysis however arise from the dynamic changes of its composition and volume. Urin and lung fluid are contributors of AF, swallowing and intramembranous transport reduce AF volume; the latter occurs at the amnion covering the placenta and allows direct exchange between AF and fetal blood. Hydrostatic and osmotic gradients additionally act on AF volume and composition. Despite these difficulties in AF reseach, available evidence allows the assumption that AF-nt-proBNP is of fetal origin [30–34].

We previously established reference intervals for AF-nt-proBNP [22]. The aim of our present study was to examine the role of AF-nt-proBNP as a biomarker in pregnancies complicated by fetal conditions associated with myocardial load.

## Material and methods

### Patients

The study was approved by the Medical School Ethics Committee of the University of Bonn (registration number 305/11). All participating women gave their written consent. Patients with gestational age (GA) between 10 and 34 weeks, presenting at our Center for Prenatal Diagnosis between November 2011 and March 2015 who were scheduled to undergo any invasive intervention that implies access to the amniotic cavity were eligible. No procedure was undertaken for the sole purpose of the study. Additionally, a subset of women who underwent scheduled cesarean delivery between 35 and 40 weeks of gestation were invited to participate. In all cases a detailed assessment of the fetal anatomy and cardiovascular status including echocardiography and Doppler examination was performed. We previously established reference values for AF-ntproBNP and extended these findings by further recruitment of controls. Classification of patients as cases and controls was undertaken according to the following criteria:

Fetuses with conditions that imply an impact on the cardiovascular function such as cardiac, thoracic and urinary tract malformations, congenital diaphragmatic hernia, tumors or intrauterine infections with alterations in cardiovascular loading, chronic TTS, FGR and hydrops were classified as cases. Fetuses with cerebral and skeletal malformations, neural tube defects, and aneuploidies without associated malformations were assigned to the control group, which otherwise consisted of fetuses without any structural abnormality. Further classification criteria were estimated fetal weight, Doppler examination of the umbilical and middle cerebral artery, and qualitative echocardiography. Maternal exclusion criteria were the presence of cardiac or renal diseases. Inclusion and exclusion criteria for controls have been described in detail [35, 36].

In a subset of patients, maternal and fetal blood samples were collected for analysis of hemoglobin and nt-proBNP concentrations. Neonatal notes were reviewed in all cases that ended in a life birth.

### Ultrasound, Doppler examination and echocardiography

A detailed description of the fetal assessment, including ultrasound technique, Doppler studies and echocardiography has been published [22]. All examinations followed a standardized protocol and were performed with high-resolution equipment by specialists in fetal diagnostic imaging.

### Sample analysis

Nt-proBNP was analyzed with a commercially available chemiluminescence immunoassay on a Dimension Vista 1500, and amniotic fluid total protein concentration (AF-TP) by photometry on a Dimension RxL Max System (Siemens Healthcare Diagnostics, Eschborn, Germany). For nt-proBNP, inter- and intra-assay coefficients of variation were 3.5% and 2.3%, respectively. All samples were processed within two hours after retrieval.

### Statistical analysis

To adjust for a potential dilutional effect, the AF-nt-proBNP / AF-TP ratio was calculated and used for analysis. The previously established reference curve was extended to incorporate samples from 34 to 40 weeks of gestation [35, 37]. Non-parametric tests were used for between group analysis of quantitative variables; Pearson’s coefficient was calculated to analyze correlations. Fetal hemoglobin values were transformed into Multiples of the Median (MoM) [38]. We used the statistical software package SPSS 22.0 (SPSS Inc., Chicago, Ill., USA) and SAS Version 9.2 (SAS Institute Inc., Cary, North Carolina, USA) for analysis.

## Results

**Fig 1:**
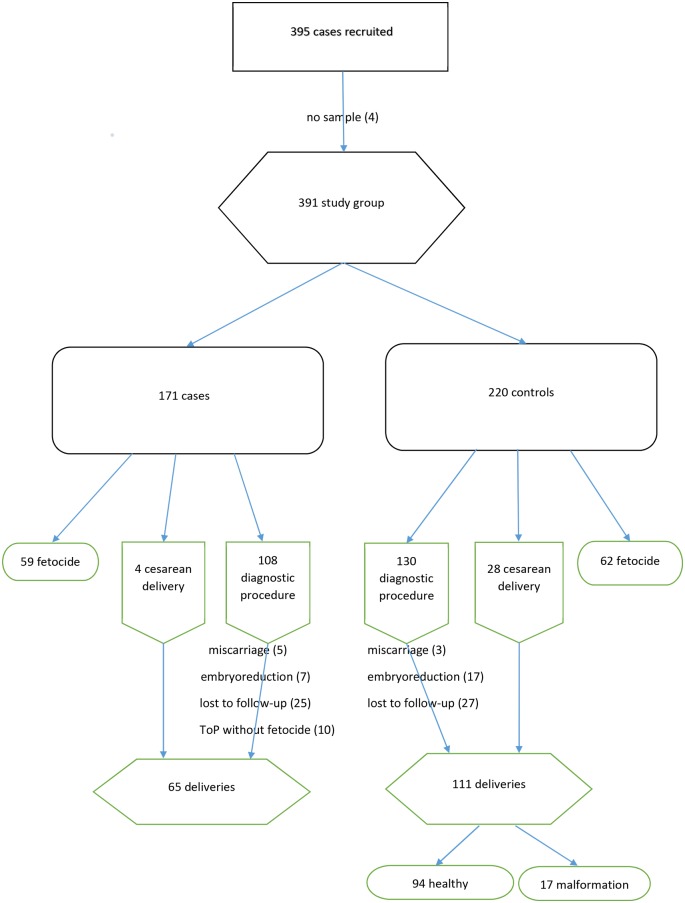
Study flow diagram.

In total, 395 samples were collected. Fig 1 illustrates the flow of the cases through the study. Clinical, obstetric and laboratory details are presented in Table 1, fetal conditions in Table 2. Multiple pregnancies were present in 59 cases. For life births, information on the outcome was retrieved from neonatal reports; prenatal diagnosis was confirmed in all cases.

**Table 1:** Clinical, obstetric and laboratory details; cases and controls.

	Group 1 (n = 171) Cases	Group 2 (n = 220) Controls	p-value
**Gestational age (weeks)**			
**Median** **IQR**	23.0 7	21.0 12	< 0.001
**Parity, n (%)**			
**0** **1–2** **≥3** **Median** **IQR**	68 (39.8) 85 (49.7) 18 (10.5) 1 2	71 (32.3) 124 (56.4) 25 (11.3) 1 2	n.s.
**Body mass index (kg/m**^**2**^**)**	(n = 100)	(n = 215)	
**Median** **Range** **IQR**	25.9 23.7 6.1	25.8 26.0 6.6	n.s.
**Indication for invasive procedure, n (%)**			
**Amniocentesis** **Fetocide / embryoreduction** **Cesarean delivery** **Amniotic fluid drainage** **Fetal blood sampling** **Intrauterine transfusion**	87 (50.9) 59 (34.5) 4 (2.3) 13 (7.6) 6 (3.5) 2 (1.2)	129 (58.6) 62 (28.2) 28 (12.7) 0 1 (0.5) 0	
**Multiple pregnancies, n**	31	28	
**Monochorionic twins** **Dichorionic twins** **Higher order**	22 7 2	0 19 9	
**Maternal hemoglobin (g/dL)**	(n = 55)	(n = 42)	
**Median** **IQR**	11.8 1.2	11.9 1.4	n.s.
MoM* **fetal hemoglobin, n (%)**	(n = 55)	(n = 40)	
**>0.84** **0.65–0.84** **0.55–0.64** **<0.55**	48 (87.3) 5 (9.1) 0 2 (3.6)	40 (100) 0 0 0	
**Maternal plasma nt-proBNP (ng/L)**	(n = 42)	(n = 34)	
**Median** **IQR**	40 27	49 44	n.s.
**Fetal plasma nt-proBNP (ng/L)**	(n = 61)	(n = 61)	
**Median** **IQR**	2628 2871	1376 1550	<0.001

*MoM, multiples of the median

**Table 2:** Fetal diagnoses at enrolment and delivery; cases and controls.

	At enrolment, n (%)	At delivery, n (%)
**Group 1, cases**	171 (100)	65 (100)
**Cardiac malformation**	27 (15.8)	10 (15.4)
syndromal disorder, nos*	25 (14.6)	6 (9.2)
**Twin-transfusion syndrome**	23 (13.5)	13 (20.0)
**Trisomy 13+18**	20 (11.7)	3 (4.6)
**Trisomy 21 with associated malformations**	15 (8.8)	2 (3.1)
**Diaphragmatic hernia**	13 (7.6)	5 (7.7)
**Hydrops**	12 (7.0)	3 (4.6)
**Fetal growth restriction**	11 (6.4)	9 (13.8)
**Tumor**	7 (4.1)	5 (7.7)
**Thoracic malformation**	6 (3.5)	4 (6.2)
**Urinary tract malformation**	4 (2.3)	0
**Others**	8 (4.7)	5 (7.7)
**Group 2, controls**	220 (100)	111 (100)
**Healthy**	122 (55.6)	94 (84.7)
**Brain malformation**	33 (15.0)	4 (3.6)
**Skeletal malformation**	17 (7.7)	6 (5.4)
**Neural tube defect**	15 (6.8)	1 (0.9)
**Trisomy 21, isolated**	13 (5.9)	2 (1.8)
Syndromal disorder, nos*	6 (2.7)	0
**Trisomy 13+18**	6 (2.7)	0
**Others**	8 (3.6)	4 (3.6)

*nos, not otherwise specified

Fetal hemoglobin and plasma nt-proBNP concentrations were determined in 95 cases (122, respectively). A very good correlation was found between nt-proBNP concentration in amniotic fluid and fetal blood (r = 0.441 for cases and r = 0.515 for controls; p < 0.01 for both). Maternal plasma nt-proBNP concentrations, analyzed in a subset of women, were within normal range. No correlation was present between nt-proBNP levels in fetal and maternal blood (maternal blood and amniotic fluid, respectively).

**Fig 2:**
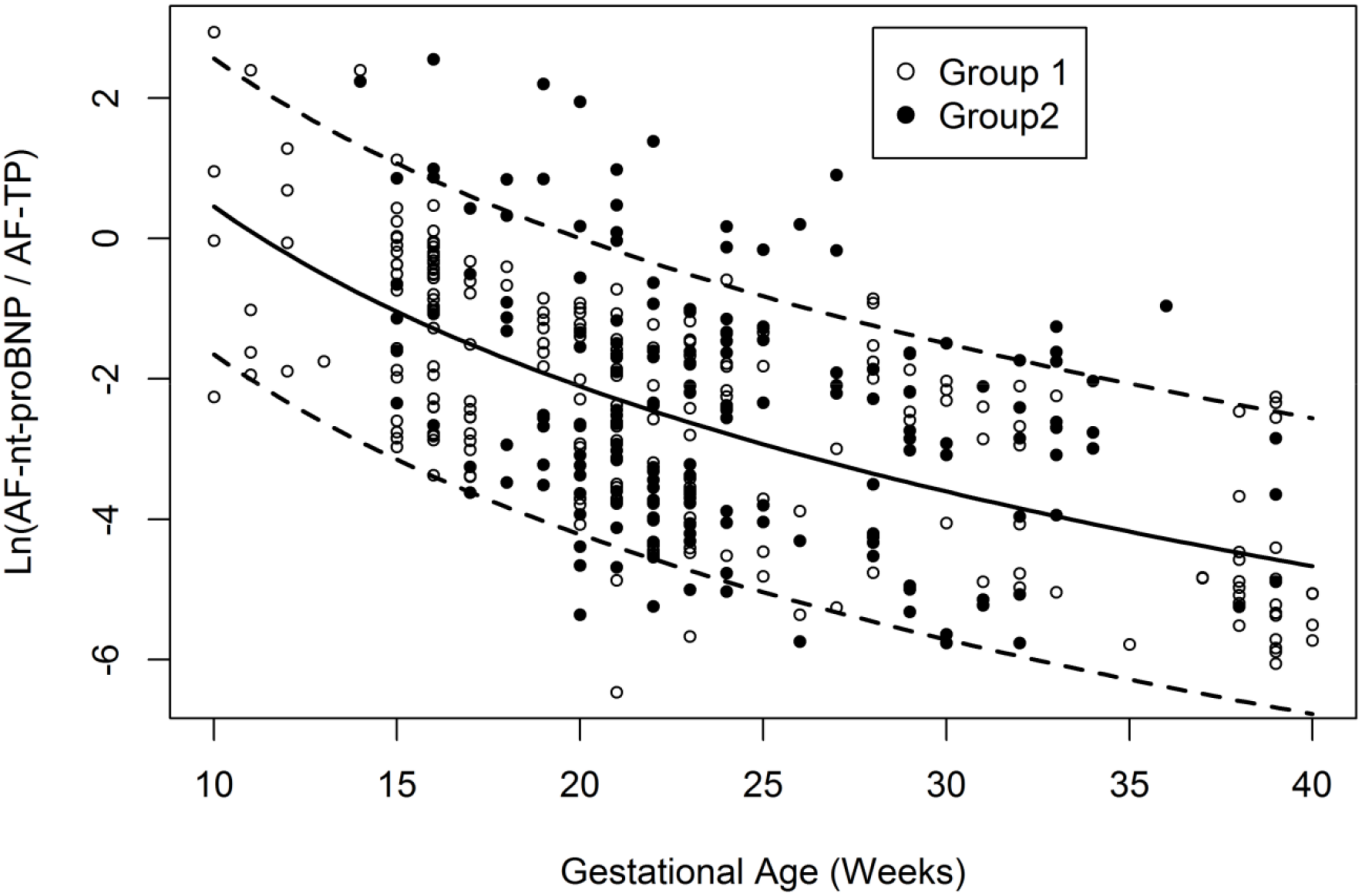
Ln (AF-nt-proBNP/AF-TP) in cases (group 1, n = 171) and controls (group 2, n = 220). Lines represent the mean and the 5% and 95% reference interval.

Fig 2 depicts the curves for the mean and 90% confidence limits of the logarithm of the AF-nt-proBNP / AF-TP ratio, with cases and controls plotted. The equation for the curve is: y = 8.965–3.696 * ln (GA). The variation around the curve was found to be constant (sd = 1.282); 5% and 95% percentile curves are calculated as mean + / - 1.282 * 1.645, the 95% quantile of the standardized normal distribution.

Specificity (0.97) and positive likelihood ratio (4.3) were high, whereas sensitivity and negative likelihood ratio were low (0.13 and 0.9, respectively).

## Discussion

We examined nt-proBNP in amniotic fluid for its role as a biomarker of myocardial dysfunction in fetal medicine, analyzing a large group of fetuses with a broad variety of disorders. The low test sensitivity precludes its application for screening purposes. However, in case of suspected myocardial dysfunction, AF-nt-proBNP determination permits confirmation. It is the only investigation in fetal medicine not based on diagnostic imaging.

Like other amniotic fluid constituents, AF-ntproBNP and AF-TP concentrations are affected by the multitude of compartments contributing to AF inflow and outflow, the hydrostatic and osmotic gradients, and the changes that occur during the course of pregnancy and in fetal disease. Although AF-ntproBNP is of fetal origin [35] we assume that these factors exert an influence on the plasma to AF ratio, act as confounders and interfere with AF analysis [30–34].

Previous investigations on AF-ntproBNP were undertaken in chronic TTS. Here, concentrations correlated with the degree of myocardial dysfunction or disease severity. However, controls were either not available or of small sample size [39–41]. A further study reported on amniotic fluid and umbilical cord BNP at delivery in monochorionic twin pregnancies, whereby higher levels were present in newborns with weight discordance >20% or myocardial dysfunction [42].

We consider AF-nt-proBNP analysis a useful adjunct method in the assessment of fetuses with myocardial dysfunction, particularly in the rapidly expanding field of minimally invasive fetal therapy, since access to the amniotic cavity is part of the procedure. Criteria for case selection, intervention timing, prognosis establishment and treatment evaluation are still being established, and are based exclusively on diagnostic imaging. AF-nt-proBNP analysis and correlation with non-invasive methods of myocardial function assessment may provide further insight into the pathophysiology of fetal cardiac function and mechanisms of myocardial adaptation.

Additionally, serial analyses of AF-nt-proBNP concentrations allow the assessment of myocardial changes after intrauterine procedures. In fetal anemia, serial analyses of circulating nt-proBNP levels revealed myocardial adaptations with potential long-term implications [23].

### Limitations

Ideally, reference intervals should be constructed with datasets from healthy cases. Furthermore, a longitudinal rather than a cross-sectional study design should be applied. However, ethical considerations prohibit an intervention with inherent complications purely for research purposes. A general limitation of nt-proBNP analysis is the assay-specificity which makes comparisons of studies performed with different assays difficult [43, 44].

### Conclusion

Indications for prenatal invasive therapy are on the rise and diagnosis, intervention timing and treatment evaluation are limited to ultrasound-based methods. AF-nt-proBNP analysis may become an additional diagnostic tool in fetal medicine. Furthermore, serial analyses may permit insight into mechanisms of myocardial adaptation that occur as a result of altered loading conditions. Nevertheless, validiation with an independent dataset is required.

## Supporting information

S1 Table Dataset. (SAV)
